# Maternal depression across childhood and offspring young adult depression and anxiety: Testing adolescent emotion dynamics as transdiagnostic mechanisms

**DOI:** 10.1002/jcv2.70130

**Published:** 2026-05-04

**Authors:** Gabrielle R. Rinne, Blakely L. Berryhill, Caroline M. Chandler, Jennifer A. Somers

**Affiliations:** ^1^ Department of Psychology University of Southern California Los Angeles California USA; ^2^ Department of Psychological Sciences Auburn University Auburn Alabama USA

**Keywords:** cumulative stress, emotion dynamics, intergenerational transmission, maternal depression, offspring mental health

## Abstract

**Background:**

Early exposure to maternal depression can increase risk for offspring mental health problems across the lifespan. Less is known about the transdiagnostic pathways through which maternal depression influences offspring mental health risk in young adulthood. This pre‐registered study tested the prospective associations of maternal depression (total exposure and instability) with offspring mental health in young adulthood and evaluated adolescent emotion dynamics as transdiagnostic mechanisms.

**Methods:**

This study used data from the Future Families and Child Wellbeing Study (FFCWS; *n* = 4898). Maternal depression was assessed when children were 1, 3, 5, and 9 years old and offspring young adult depression and anxiety was assessed at age 22 with structured clinical interviews. Adolescent daily and biweekly positive and negative emotions were assessed in two Future of Families and Child Wellbeing study substudies (*n* range = 513‐1049) when offspring were 15 years old. Informed by theory and past research, we calculated variability, instability, and inertia to assess emotion dynamics and instability to assess maternal depression dynamics. We tested study aims using logistic regression, multivariate regression, and mediation models.

**Results:**

Total exposure to maternal depression predicted greater odds of offspring young adult depression whereas instability in maternal depression was not directly associated with offspring mental health. Offspring biweekly emotion dynamics during adolescence significantly predicted subsequent anxiety and depression. Additionally, greater instability in maternal depression was associated with lower biweekly instability in sadness during adolescence, which was in turn associated with greater likelihood of young adult anxiety. Associations were independent of mean emotion levels and covariates.

**Conclusion:**

Overall, findings highlight the importance of considering how the dynamics of mood and emotions across generations (e.g., mother, offspring) and timescales (e.g., daily, biweekly, yearly) may shape young adult psychopathology. To build from these initial findings, future studies could investigate these processes using genetically informative longitudinal designs, causal mediation analyses, and continuous measures of maternal depression.

## Key Points

**What's known?**

- Exposure to maternal depression across childhood increases offspring risk for psychopathology, but less is known about how the course of maternal depression over time influences offspring risk and about the transdiagnostic mechanisms of risk.

**What's new?**

- The current study tested associations of maternal depression (total exposure and instability) with offspring young adult internalizing disorders and examined offspring emotion dynamics in adolescence as transdiagnostic mechanisms.
- Greater total exposure to maternal depression from infancy to middle childhood predicted greater odds of offspring depression in young adulthood.
- Offspring emotion dynamics during adolescence predicted young adult depression and anxiety.

**What's relevant?**

- Findings underscore the importance of considering the implications of dynamics of emotion and mood, across generations and timescales, for young adult psychopathology risk.

## INTRODUCTION

Exposure to maternal depression poses a two‐to fourfold risk of offspring mental health problems in early adulthood (Beardslee et al., 2011; Chiang et al., 2023; Van Santvoort et al., 2015). Although the adverse offspring outcomes associated with maternal depression are well‐documented (e.g., Ashman et al., 2008; Goodman, 2020; Kingston et al., 2018; Rinne et al., 2022), less work has examined the course of maternal depression spanning key developmental periods (e.g., infancy, toddlerhood, childhood). This is a notable research gap because depression is commonly understood to be a highly recurrent condition whereby individuals with depression often experience more than one depressive episode in their lifetime (Solomon et al., 2000). At the same time, the dynamics of maternal depression over time uniquely shapes offspring risk for mental health problems, even after accounting for total exposure to maternal depression (Cents et al., 2012; Glynn et al., 2018; Park et al., 2018). Moreover, the mechanisms through which maternal depression influences offspring mental health have not been thoroughly prosecuted (Glynn et al., 2018; Goodman, 2020). To address these knowledge gaps, the current study tested prospective associations of maternal depression (total exposure and instability) from infancy through middle childhood with offspring depression and anxiety in young adulthood and tested offspring emotion dynamics in adolescence as putative pathways of risk transmission.

Contrasting theories have been put forth to explain how maternal depression shapes offspring mental health risk. Existing research has been largely guided by the cumulative stress model, which argues that children who are exposed to high levels of stress, such as maternal depression, over time are at greater risk for poor developmental outcomes (Shonkoff et al., 2012). Conversely, theories of environmental unpredictability posit that the dynamics of environmental conditions over time, such as instability or variability in caregiver mental health, are unique stressors associated with offspring emotional development (see Table 1 for further detail; Davis & Glynn, 2024). For example, a growing body of literature demonstrates patterns of maternal mood that are more unpredictable, variable, or unstable from the prenatal period through early childhood distinctly influences offspring adjustment, including emotional well‐being in infancy, childhood, and adolescence (for review, see Davis & Glynn, 2024). To our knowledge, however, no studies have compared the prospective associations of maternal depression total exposure and maternal depression dynamics across infancy, toddlerhood, and childhood with offspring mental health in young adulthood. Furthermore, critical knowledge gaps remain as to the mechanisms through which total exposure to and the dynamics of maternal depression sculpt offspring adjustment.

**TABLE 1:** Overview of emotion and environmental dynamics.

Dynamic	Definition	Common statistical operationalizations	Empirical example: Emotion dynamics	Empirical example: Maternal depression
Variability	Variability over time	Within‐person standard deviation (iSD)	Within‐person standard deviation of positive and negative affect in experience‐sampling studies (Houben et al., 2015; Kuppens et al., 2010)	Within‐mother variability in depressive symptoms over time (Halligan et al., 2007; Vliegen et al., 2014)
Instability	The tendency to fluctuate from one moment to the next	Mean square successive difference (MSSD) or related metrics	MSSD of momentary affect indexing emotional instability from one moment to the next (Jahng et al., 2008; Koval et al., 2013)	Instability in maternal depression indexed via successive changes across repeated assessments (Cents et al., 2012; van der Waerden et al., 2015)
Inertia	Resistance to change over time	Autocorrelation over time	Lagged autocorrelation of affect indicating persistence of emotional states over time (Houben et al., 2015; Kuppens et al., 2010; Shao & Ong, 2026)	Persistence of maternal depressive symptoms across time, reflected in strong temporal autocorrelations or chronic symptom trajectories (Campbell et al., 1995; Goodman et al., 2011)

*Note*: Dynamics can be examined across negative and positive valences and timescales (e.g., hourly, daily, weekly) and levels of analysis (e.g., emotions, environmental measures).

Adolescent emotion dynamics may be transdiagnostic mechanisms of the intergenerational transmission of maternal depression. Prior evidence has shown that maternal depression influences offspring emotion‐related processes through interrelated biological (e.g., altered emotion‐related neurodevelopment), psychological (e.g., modeling of emotion regulation), and social pathways (e.g., attachment) (Ashman et al., 2008; Buckholdt et al., 2013; Buckingham‐Howes et al., 2017; Glynn et al., 2018; Goodman et al., 2020; Sabalbal et al., 2025; Stone & Sylvester, 2025; Van Santvoort et al., 2015). Beyond mean levels of emotions, emotion dynamics (see Table 1; for review, Houben et al., 2015) may be cause and consequence of dysregulated emotion processes and as such represent novel transdiagnostic risk markers (Houben et al., 2015; Kuppens et al., 2010; McKone & Silk, 2022). For example, seminal theories on emotion regulation (e.g., Gross' 1998 process model of emotion regulation) contend that how emotions unfold over time, over and above mean levels of emotion, are central to well‐being and psychological functioning (see Shao & Ong, 2026 for a review). Consistent with such theories, meta‐analytic evidence demonstrates that more variable, less stable, and more inert emotions, particularly negative emotions, predict lower levels of psychological well‐being and greater depressive symptoms (Houben et al., 2015).

To date, most studies examining associations of emotion dynamics with psychopathology have been conducted in adult samples (Houben et al., 2015; McKone & Silk, 2022). Further attention to these processes early in development may be useful to identify antecedents of transdiagnostic psychopathology. Adolescence may be a particularly important period to examine emotion dynamics, as it is a developmental period often accompanied by heightened emotional lability (McKone & Silk, 2022) and risk for internalizing problems (Buckholdt et al., 2013; Ip et al., 2021). A few recent studies suggest that adolescent emotional dynamics may portend risk for mental health problems at later timepoints. For example, promising work among at‐risk samples (based on maternal depression history) suggests that less rigidity in positive emotions prospectively predicted fewer future depressive symptoms (Fisher et al., 2025). Likewise, even after accounting for mean levels of affect, greater inertia in adolescent positive affect was associated with adolescent depression diagnosis (Abitante et al., 2024). However, although one recent meta‐analysis reported that heightened variability in adolescent emotions were associated with youth mental health problems, the nature of the association between emotion dynamics and adolescent mental health differed depending on the measure of dynamics and discrete emotion under investigation (Reitsema et al., 2022). Further, relative to work on adult emotion dynamics, the nascent literature on adolescent dynamics has paid less attention to temporally sensitive dynamics versus slower‐unfolding changes in mood (Hollenstein, 2015; Reitsema et al., 2022). Critically, emotion dynamics across timescales may represent different constructs that differentially relate to psychopathology (e.g., day‐to‐day variation may reflect responsiveness to situational contingencies whereas longer timescales may reflect “mood swings”) (Bos et al., 2019; Koval et al., 2016).

In addition to the paucity of research on adolescent emotion dynamics across timescales, only a few studies to our knowledge have examined the environmental factors that shape adolescent emotion dynamics, and with mixed results. In one study, adverse childhood experiences (ACEs), including exposure to parental psychopathology, predicted greater adolescent positive emotional variability (Pelotonen et al., 2024). However, as ACEs have been conceptualized from both cumulative stress and unpredictability frameworks, it is not clear how distinct characteristics of total exposure to and dynamics of maternal depression would affect offspring emotion dynamics during adolescence. Moreover, another study reported null concurrent associations of maternal depression with adolescent emotional variability or inertia (Abitante et al., 2024). Thus, further research is necessary clarify the role maternal depression plays in sculpting offspring emotion dynamics.

### Current study aims

The purpose of the present study was to evaluate prospective associations of total exposure to maternal depression and instability in maternal depression on offspring depression and anxiety in adulthood and test emotion dynamics as a pathway. We tested study aims among a sample of predominantly low‐income, ethnic minority families enrolled in the Future of Families and Child Wellbeing study (FFCWS) and two FFCWS substudies. Addressing gaps in the literature that has predominantly focused on day‐to‐day emotion dynamics (Reitsema et al., 2022), we evaluated our hypotheses across two salient timescales: day‐to‐day (Study 1) and biweekly dynamics (Study 2).

**FIGURE 1:**
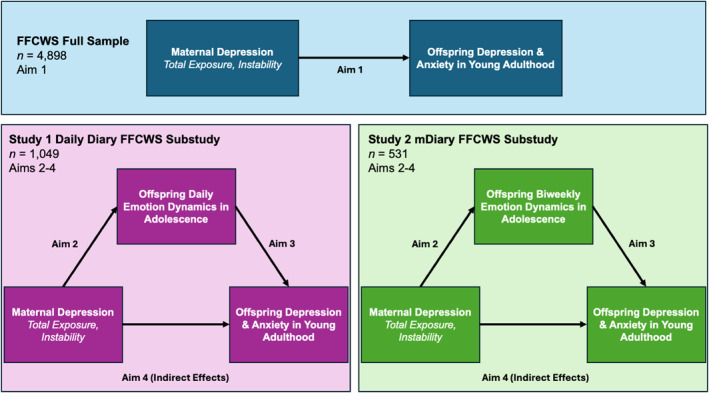
Conceptual overview of study aims.

Figure 1 presents a conceptual overview of the present study. We first tested the hypothesis that youth who experienced more variable exposure to maternal depression from infancy through early childhood would have a greater likelihood of experiencing depression and anxiety in early adulthood in the full FFCWS sample (Aim 1). In each substudy, we tested three additional hypotheses. Specifically, we tested the hypothesis that adolescents who experienced greater overall exposure and more unstable exposure to maternal depression would exhibit greater emotional variability, instability, and inertia (Aim 2). In turn, we predicted that emotional variability, instability, and inertia would be positively associated with depression and anxiety in young adulthood (Aim 3). Finally, we tested the hypothesis that maternal depression would influence young adult depression and anxiety indirectly via greater adolescent day‐to‐day and biweekly emotional variability, instability, and inertia (Aim 4).

## METHOD

### Participants and procedure

The current study used data from the Future of Families and Child Wellbeing (FFCWS) study, a longitudinal birth cohort study that assessed 4898 caregivers and children with a high proportion of mothers from racial and ethnic minority backgrounds and lower socioeconomic status (see Reichman et al., 2001 for further detail on study design and cohort characteristics). Families were recruited from local hospitals at the time of the child's birth. Mothers completed questionnaires and structured clinical interviews in the FFCWS over five follow‐up waves, when children were approximately 1, 3, 5, 9, and 15 years of age. Youth were interviewed at ages 9, 15, and 22 years. At the year‐15 wave, primary caregivers completed questionnaires and structured clinical interviews; most primary caregivers were biological mothers (87.9%).

Emotion dynamics were assessed in two substudies of FFCWS, the *mDiary* and a sleep study, each conducted around the year‐15 follow‐up wave (see below for further detail). Sample characteristics of participants in each substudy are shown in Table 2.

**TABLE 2:** Sample characteristics.

	Full sample *n* = 4898	Study 1: Daily diary *n* = 1049	Study 2: mDiary *n* = 531
	*M* (SD) or % (*n*)	*M* (SD) or % (*n*)	*M* (SD) or % (*n*)
Child biological sex (% male)	47.8% (2341)	47.7% (427)	44.8% (238)
Poverty ratio	2.22 (2.41)	2.40 (2.55)	3.02 (2.92)
% Married to child's father at enrollment	24.2% (1187)	23.2% (333)	34.1% (181)
Mother age at enrollment (years)	25.28 (6.04)	25.24 (5.88)	26.28 (6.29)
Mother educational attainment			
Less than high school	34.7% (1699)	28.9% (259)	23.5% (125)
High school or equivalent	30.2% (1480)	32.8% (294)	27.9% (148)
Some college or technical school	24.3% (1189)	27.1% (243)	31.5% (167)
College or graduate school	10.7% (524)	10.9% (98)	17.1% (91)
Mother race and ethnicity			
White, non‐Hispanic	21.0% (1030)	21.7% (194)	31.6% (168)
Black, non‐Hispanic	47.7% (2326)	46.3% (415)	35.4% (188)
Hispanic	27.3% (1336)	28.0% (251)	28.6% (152)
Other	4.0% (194)	4.0% (36)	4.1% (22)

*Note*: Poverty ratio was calculated as the ratio of household income to poverty threshold.

#### Study 1: Sleep study

The sleep study recruited a randomly selected subset of adolescents who participated in the year‐15 wave of FFCWS (*N* = 1049). Recruitment occurred between February 2014 and March 2016. In the sleep study, adolescents were asked to complete a diary each evening after 7:00 PM and before going to sleep, such that the questions in the diary asked about the current day, for seven consecutive days (*T* = 7; see Mathew et al., 2023 for additional details). Diaries were administered online via Qualtrics. Participants were compensated for completing the diaries.

#### Study 2: mDiary study

The mDiary study recruited a subset of the year‐15 participants to participate in online bi‐weekly surveys over 52 weeks of FFCWS (*T* = 26 diaries; *N* = 531; see Goldberg et al., 2019; Tienda et al., 2022 for more details). Recruitment for the mDiary study occurred between November 2015–April 2017, approximately one year after the year‐15 study visit in the FFCWS study. Most participants completed bi‐weekly surveys on mobile devices (85%; described further in Goldberg et al., 2019). Participants were compensated for each survey they completed. Of the 531 participants in the mDiary study, 219 also participated in the sleep study.

### Measures

#### Maternal depression from 1 to 9 years

Mothers completed the Composite International Diagnostic Interview Short Form (CIDI‐SF; Kessler et al., 1998), a structured clinical interview, at each of the first four waves of the FFCWS (child age 1, 3, 5, and 9). The CIDI‐SF is a standardized instrument for the assessment of mental disorders, with questions consistent with the Diagnostic and Statistical Manual of Mental Disorders—Fourth Edition (DSM‐IV). Mothers reported on whether, in the past year, they were unable to enjoy things they normally found enjoyable or experienced feelings of depression. Mothers who reported experiencing at least one of those symptoms most of the day, every day for at least a 2‐week period were asked seven additional symptom questions (e.g., trouble sleeping or concentrating, feeling tired or worthless). Consistent with prior work in the FFCWS sample (e.g., Meadows et al., 2010; Turney, 2011), mothers who endorsed three or more of the symptoms met liberal criteria for depression within the last 12 months of each wave. This approach allows for greater sensitivity to detect depression in community samples and is appropriate for use in epidemiological, cross‐cultural, and other research studies (Kessler et al., 1998).

#### Offspring emotions in adolescence (15 years)

Items assessing offspring emotions during adolescence were developed from the PANAS in the sleep study (Study 1) and developed by the FFCWS team for the mDiary study (Study 2) (see Mathew et al., 2023 for further detail).

##### Positive emotions

Happiness was assessed in both studies. In the sleep study (Study 1), participants were asked to report how happy they felt that day, with options ranging from 1 (*very slightly or not at all*) to 5 (*extremely*). In the mDiary study (Study 2), participants were asked to report how often they felt happy in the past 2 weeks, with options ranging from 1 (*never or rarely*) to 4 (*most of the time*).

##### Negative emotions

Negative emotions were assessed in each study, although the discrete negative emotions differed across the studies. In the sleep study (Study 1), participants were asked to report on how lonely and how angry they felt that day, with options ranging from 1 (*very slightly or not at all*) to 5 (*extremely*). In the mDiary study (Study 2), participants were asked to report how often they felt sad in the past 2 weeks, with options ranging from 1 (*never or rarely*) to 4 (*most of the time*).

#### Offspring depression and anxiety in young adulthood (22 years)

Offspring completed the CIDI‐SF (Kessler et al., 1998), a structured clinical interview, at the year 22 follow‐up wave. The present study used constructed variables that indicated whether the respondent met liberal criteria for major depression in the last 12 months and whether they met anxious criteria in the last 12 months.

### Data analytic plan

#### Preliminary analyses

##### Operationalization of maternal depression total exposure and instability

About 14% of mothers met criteria for depression when children were 1 year old (13.8%), 17.7% when children were 3 years old, 14.3% when children were 5 years old, and 12.5% when children were 9 years old. Maternal depression was operationalized two ways: total exposure and instability.

We used a descriptive approach to characterize total exposure to maternal depression from child ages 1–9 years, informed by both the cumulative stress model (Shonkoff et al., 2012) and past research. This approach was previously employed by past studies using FFCWS data that characterized trajectories of parental behavior and involvement (Geller & Curtis, 2018). We used this descriptive approach because missingness on depression was related to depression diagnostic status at future time points (e.g., mothers missing on 9‐year depression were more likely to be depressed between 9 and 15 years, *χ* ^2^(1) = 3.950, *p* = 0.047); therefore, a pure count variable would bias the data toward never‐depressed mothers. In contrast, the descriptive approach takes into account the relationship between depression and study attrition in a graduated approach. Please see Supporting Information S1: Appendix S1, Table S1 for a further detail and description of the total exposure categories for maternal depression.

To assess the dynamics of maternal depression over time, we calculated instability in maternal depression as the mean square of successive differences (MSSD). Past work has operationalized dynamics of maternal depression over time using instability (MSSD), variability (intra‐individual standard deviation [iSD]), and inertia (see Table 1 for further detail and empirical examples); given that maternal depression was a binary measure across timepoints, the present study focused on instability in maternal depression. Maternal depression total exposure and instability in maternal depression were significantly positively correlated (*r* = 0.69, *p* < 0.001). The positive correlation between maternal mood instability and total exposure aligns with related work on maternal mood unpredictability (Glynn et al., 2018).

##### Operationalization of emotion dynamics

We calculated three aspects of emotion dynamics (variability, instability, and inertia) per emotion variable in each study, consistent with past theory‐driven studies on emotion dynamics (for a meta‐analysis using these operational definitions, see Houben et al., 2015; see also Table 1 for definitions and empirical examples). Given that systematic trends in affect time series data would confound interpretation of emotion dynamics (especially variability and autocorrelation; Jahng et al., 2008), we first evaluated each time series for statistically significant linear trends using SAS Proc Mixed; if such trends were present, the time series were detrended and emotion dynamics were calculated on the detrended data (see Supporting Information S1: Appendix S2 for more details).

##### Emotional variability

Emotional variability was calculated as the iSD of each affect time series, per participant.

##### Emotional instability

Emotional instability was calculated as the MSSD of each affect time series, per participant.

##### Emotional inertia

Emotional inertia was calculated as the autocorrelation (at a lag‐1) in each affect time series, per participant, using SAS Proc Autoreg (SAS Institute Inc, 2014).

#### Primary analyses

Primary regression analyses were conducted within a structural equation modeling framework using M*Plus* version 8.8 (Muthén & Muthén, 1998‐2017). We first tested associations of maternal depression (total exposure and instability) with young adult depression and anxiety (Aim 1) in the full FFCWS sample with logistic regression, adjusting for covariates. Aims 2‐4 were tested separately in Study 1 and Study 2. Aim 2 tested associations of maternal depression (total exposure and instability) with adolescent emotion dynamics at age 15 using multivariate regression models. Maternal depression total exposure and instability were tested in the same models for Aims 1 and 2 to estimate unique statistical effects of each measure on young adult outcomes and adolescent emotion dynamics, respectively. Aim 3 tested associations of adolescent emotion dynamics at age 15 with young adult depression and anxiety, using logistic regression.

To test Aim 4, we conducted a structural equation model that modeled paths for the statistical effects of maternal depression on adolescent emotion dynamics (a’ path) and adolescent emotion dynamics on young adult depression and anxiety (b’ path). We then estimated the indirect effect of maternal depression on young adult outcomes via adolescent emotion dynamics with RMediation (Tofighi & MacKinnon, 2011). RMediation produces 95% confidence intervals (CIs) of the indirect effect based on the distribution of the product and an asymptotic normal distribution. Evidence of an indirect effect of maternal depression on offspring depression and anxiety during young adulthood via adolescent emotion dynamics exists if the 95% CI of the indirect effect does not include zero. Our approach to testing indirect effects in Aim 4 was informed by current expert recommendations in mediation analyses (e.g., O’Rourke & MacKinnon, 2015; Rijnhart et al., 2021) and characteristics of the data that precluded other methods of testing indirect effects, such as causal mediation analyses that require binary predictors and repeated assessments of predictors, mediators, and outcomes. Indirect effects may be present even in the absence of significant direct effects and can have greater power than tests of direct effects (MacKinnon et al., 2000, 2002).

In all analyses, we employed maximum likelihood estimation with robust standard errors (MLR) to account for non‐normality (e.g., some emotion dynamics were skewed and/or kurtotic). As expected, measures of emotion dynamics were moderately to strongly correlated within emotions in both studies; therefore, consistent with prior work, each measure was tested in separate models to avoid collinearity (c.f. Abitante et al., 2024; see Supporting Information S1: Appendix S2 for further detail). Following recommendations in the ecological momentary assessment (EMA) literature (e.g., Griffiths et al., 2022), primary analyses for Aims 2‐4 used emotion dynamics for participants who completed at least 4/7ths of the diaries (see below for detail on secondary analyses with full sample). Across emotion dynamics and substudies, approximately 70.0% of sample (range: 70.0%–74.1%) were included in the “primary” sample; we also included covariates related to missingness (detailed below; for more information on demographic correlates of completion rates, please see Tienda et al., 2023).

Depending on the analysis, in the main text we present the *p*‐values and either odds ratio (OR) and 95% CI of the OR (for logistic regression), or the unstandardized coefficient (B), standard error of the unstandardized coefficient, and standardized coefficients (for multivariate linear regression). To avoid redundancy with main text, tables present the ORs and their standard errors, or the standardized coefficients and their standard errors. Significance was determined based on an alpha level of 0.05, and “significantly” is used in primary results to refer to statistically significant associations that exceeded the threshold of statistical significance (*p* < 0.05).

##### Covariates

Primary analyses adjusted for covariates, selected for inclusion based on associations with primary study variables and/or missingness on primary study variables to satisfy the assumptions of the estimator (see Supporting Information S1: Appendix S3). Covariates included in analytic models were: maternal race (white, non‐Hispanic; black, non‐Hispanic; Hispanic; other), child biological sex, baseline household poverty ratio the ratio of total household income to the official poverty thresholds, designated by the U.S. Census Bureau, for the year preceding the baseline interview), maternal education (less than high school, high school or equivalent, some college or technical/trade school, college or graduate degree), maternal marital status. The number of surveys completed and mean levels of each emotion (for each time series, per participant) were obtained and included as covariates in primary analyses in Aim 2 and 3.

#### Secondary analyses

We conducted three sets of secondary analyses. First, we conducted a robustness analysis that included maternal depression between 9 and 15 years (based on the CIDI‐SF liberal criteria) and youth internalizing at the year 15 wave (based on mean of primary caregiver report of youth depression and anxiety problems on a 34‐item version of the CBCL; alpha = 0.79) as additional covariates. Second, we examined whether the pattern of results varied in the primary analytic sample who completed 4/7^ths^ of the diaries versus the full sample. Third, we tested the associations of maternal depression total exposure and instability with offspring depression and anxiety (Aim 1) in the Study 1 and Study 2 samples separately.

### Transparency and openness

Our aims and hypotheses were preregistered (https://osf.io/5s7kb). Deviations from preregistration were due to data availability and data properties, described in detail in the Supporting Information file (Supporting Information S1: Appendix S1, Table S2). Sample size was determined by the parent study. Published power analyses suggest that 539 participants are needed to achieve 0.8 power to detect a significant indirect effect when both paths involved are small using the asymptotic product of the CIs (Fritz & MacKinnon, 2007); thus, we are sufficiently powered to test our primary aims. Data are publicly available by request from the FFCWS team. We report how we determined our sample size, all data exclusions, all manipulations, and all measures in the study. The analytic code necessary to reproduce the primary analyses is available by request from the first author.

## RESULTS

### Descriptive statistics

Table 3 presents descriptive statistics of primary study variables in the full sample, Study 1, and Study 2. Supporting Information S1: Table S3 presents bivariate correlations for the emotion dynamics measures across Study 1 and Study 2.

**TABLE 3:** Descriptive statistics of primary study variables.

	*M* (SD)	Range
Full sample		
Maternal depression		
Total Exposure	2.20 (1.21)	1–6
Instability	0.22 (0.29)	0–1
Study 1		
Maternal depression		
Total Exposure	2.13 (1.28)	1–6
Instability	0.21 (0.29)	0–1
Daily emotion dynamics		
Happy iSD^a^	1.10 (0.06)	0.98–1.26
Happy MSSD^a^	2.82 (1.05)	0.00–7.30
Happy Inertia	0.22 (0.31)	−0.78–0.97
Angry iSD	0.60 (0.55)	0.00–2.31
Angry MSSD	1.25 (2.03)	0.00–16.00
Angry Inertia	0.16 (0.30)	−0.91–0.95
Lonely iSD	0.47 (0.54)	0.00–2.83
Lonely MSSD	0.90 (1.79)	0.00–16.00
Lonely Inertia	0.14 (0.29)	−0.91–0.96
Study 2		
Maternal depression		
Total Exposure	2.01 (1.38)	1–6
Instability	0.18 (0.27)	0–1
Biweekly emotion dynamics		
Happy iSD^a^	1.04 (0.05)	1.00–1.27
Happy MSSD^a^	2.07 (0.78)	0.01–5.20
Happy Inertia	0.01 (0.27)	−0.70–0.89
Sad iSD^a^	1.04 (0.04)	1.00–1.23
Sad MSSD^a^	2.07 (0.74)	0.32–4.80
Sad Inertia	0.02 (0.26)	−0.94–0.78

*Note*: iSD, Variability; MSSD, Instability.

^a^ Emotion dynamics were calculated on the detrended time series.

***p < 0.001; **p < 0.01; *p < 0.05; ^*p* < 0.10.

#### Full sample

In the full sample, 12.7% of young adults met criteria for anxiety and 38.6% met criteria for depression. In independent samples *t*‐tests (see Supporting Information S1: Table S4), young adults who met criteria for depression had greater total exposure to maternal depression and to greater instability in maternal depression compared to young adults who did not. Similarly, young adults who met criteria for anxiety at age 22 had greater total exposure to maternal depression and instability in maternal depression compared to young adults who did not meet criteria for anxiety (Supporting Information S1: Table S4).

#### Study 1 (daily diary)

11.9% of young adults met criteria for anxiety, and 38.6% met criteria for depression in Study 1. In bivariate correlations, maternal depression total exposure and instability were not significantly associated with offspring daily emotion dynamics in adolescence. As shown in Supporting Information S1: Table S4, in Study 1, offspring with anxiety in young adulthood had significantly greater daily variability and inertia in anger and greater daily variability, instability, and inertia in loneliness. Similarly, offspring with depression in young adulthood had significantly greater daily variability and instability in anger and significantly greater daily instability and inertia in sadness in Study 1 (Supporting Information S1: Table S4).

#### Study 2 (mDiary)

17.5% of young adults met criteria for anxiety, and 45.8% met criteria for depression in Study 2. Maternal depression total exposure and instability were not significantly associated with offspring biweekly emotion dynamics in adolescence. Young adults with depression had significantly lower biweekly instability and inertia in happiness (Supporting Information S1: Table S4).

### Primary results^1^

#### Aim 1: Associations of maternal depression with young adult depression and anxiety

In covariate‐adjusted models conducted among full sample, total exposure to maternal depression significantly predicted young adult depression (OR = 1.10, 95% CI [1.01, 1.20], *p* = 0.025) and was also non‐significantly associated with young adult anxiety (OR = 1.13, 95% CI [1.00, 1.29], *p* = 0.062). Instability of maternal depression was not significantly associated with young adult depression (OR = 1.40, 95% CI [0.93, 2.12], *p* = 0.107) or anxiety (OR = 1.21, 95% CI [0.64, 2.26], *p* = 0.556).

#### Aim 2: Associations of maternal depression with adolescent emotion dynamics

Table 4 presents complete regression coefficients for Aim 2 analyses.

**TABLE 4:** Associations of maternal depression and offspring emotion dynamics in adolescence (Aim 2).

	Happy			Lonely			Angry		
	iSD	MSSD	Inertia	iSD	MSSD	Inertia	iSD	MSSD	Inertia
	β (SE)	β (SE)	β (SE)	β (SE)	β (SE)	β (SE)	β (SE)	β (SE)	β (SE)
Study 1: Maternal depression → adolescent daily emotion dynamics									
Maternal depression: Total exposure	−0.012 (0.041)	0.064 (0.053)	0.092 (0.050)^	0.006 (0.037)	0.021 (0.039)	−0.013 (0.063)	−0.018 (0.041)	−0.013 (0.045)	−0.074 (0.074)
Maternal depression: Instability	−0.014 (0.045)	−0.113 (0.059)*	−0.097 (0.065)^	0.024 (0.035)	0.011 (0.038)	0.058 (0.059)	0.011 (0.039)	−0.033 (0.042)	0.036 (0.045)

	Happy			Sad		
	iSD	MSSD	Inertia	iSD	MSSD	Inertia
	β (SE)	β (SE)	β (SE)	β (SE)	β (SE)	β (SE)
Study 2: Maternal depression → adolescent biweekly emotion dynamics						
Maternal depression: Total exposure	0.013 (0.051)	0.027 (0.064)	−0.002 (0.068)	0.042 (0.043)	0.096 (0.045)*	0.096 (0.057)^
Maternal depression: Instability	−0.057 (0.046)	−0.008 (0.062)	0.016 (0.067)	−0.088 (0.042)*	−0.121 (0.054)*	−0.101 (0.067)

*Note*: iSD, variability; MSSD, instability. β, standardized coefficient. SE, standard error of the standardized coefficient. Models adjusted for maternal race, child biological sex, poverty ratio, maternal education, maternal marital status, the number of surveys completed, and mean levels of each emotion. For ease of reading, covariate effects are not presented (see Supplemental Materials for a description of covariate effects).

*p < 0.05; ^*p* < 0.10.

##### Study 1 (daily diary)

Instability of maternal depression predicted lower daily instability (*B* = −0.459, SE = 0.234, *p* = 0.050, *β* = −0.113) in adolescent happiness. Neither maternal depression total exposure nor instability predicted daily dynamics of adolescent loneliness or anger (*p*'s > 0.067).

##### Study 2 (mDiary)

Instability of maternal depression predicted significantly lower biweekly instability (*B* = −0.351, SE = 0.155, *p* = 0.024, *β* = −0.121) and variability (*B* = −0.016, SE = 0.008, *p* = 0.036, *β* = −0.088) in adolescent sadness. In contrast, total exposure to maternal depression was associated with significantly greater biweekly instability (*B* = 0.059, SE = 0.028, *p* = 0.031, *β* = −0.096) in adolescent sadness. Maternal depression did not predict biweekly dynamics of adolescent happiness.

#### Aim 3: Associations of adolescent emotion dynamics with young adult depression and anxiety

Table 5 presents complete regression coefficients for Aim 3 analyses.

**TABLE 5:** Associations of adolescent emotion dynamics with young adult depression and anxiety (Aim 3).

	Depression	Anxiety
	OR (SE)	OR (SE)
Study 1: Adolescent daily emotion dynamics → young adult depression and anxiety		
Happy		
iSD	1.313 (2.174)	0.522 (1.320)
MSSD	0.944 (0.084)	1.086 (0.166)
Inertia	1.041 (0.242)	1.801 (0.698)
Lonely		
iSD	1.365 (0.312)	2.029 (0.789)
MSSD	0.991 (0.055)	1.079 (0.078)
Inertia	1.424 (0.385)	2.207 (1.090)
Angry		
iSD	1.180 (0.263)	1.250 (0.447)
MSSD	1.004 (0.053)	0.960 (0.085)
Inertia	1.125 (0.285)	2.218 (0.955)
Study 2: Adolescent biweekly emotion dynamics → young adult depression and anxiety		
Happy		
iSD	0.012 (0.055)	0.018 (0.035)*
MSSD	0.660 (0.090)**	0.606 (0.135)*
Inertia	0.322 (0.125)**	0.524 (0.291)
Sad		
iSD	0.037 (0.076)	0.021 (0.106)
MSSD	0.833 (0.113)	0.558 (0.141)*
Inertia	0.729 (0.269)	0.408 (0.246)

*Note*: iSD, variability; MSSD, instability. Models adjusted for maternal race, child biological sex, poverty ratio, maternal education, maternal marital status, the number of surveys completed, and mean levels of each emotion. For ease of reading, covariate effects are not presented (see Supplemental Materials for a description of covariate effects).

**p < 0.01; *p < 0.05; ^*p* < 0.10.

##### Study 1 (daily diary)

Daily dynamics of adolescent happiness, anger, and loneliness were not associated with young adult depression or anxiety (*p*'s > 0.064). However, mean levels of anger, loneliness, and happiness each predicted significantly higher likelihood of young adult anxiety (OR's = 1.63–1.76, *p*'s = 0.01‐0.05). Mean levels of anger and loneliness also predicted greater likelihood of young adult depression (OR's = 1.44–1.58, *p*'s < 0.001‐0.01).

##### Study 2 (mDiary)

Biweekly adolescent emotion dynamics were associated with young adult depression and anxiety. Greater instability (OR = 0.660, 95% CI [0.506, 0.861], *p* = 0.002) and inertia (OR = 0.322, 95% CI [0.150, 0.688], *p* = 0.003) in adolescent happiness each significantly predicted decreased odds of young adult depression. Variability (OR = 0.018, 95% CI [0.000, 0.890], *p* = 0.044) and instability in adolescent happiness (OR = 0.606, 95% CI [0.392, 0.938], *p* = 0.024) also predicted significantly decreased odds of young adult anxiety. Similarly, instability in adolescent sadness predicted significantly decreased odds of young adult anxiety (OR = 0.558, 95% CI [0.340, 0.916], *p* = 0.021). Neither variability and inertia in adolescent sadness were significantly associated with young adult depression or anxiety (*p*'s > 0.110).

#### Aim 4: Indirect effect of maternal depression on young adult depression and anxiety via adolescent emotion dynamics

In Study 1, there was no evidence of an indirect effect of maternal depression on young adult depression and anxiety via daily dynamics of adolescent happiness, anger, and loneliness.

In Study 2, there was evidence that instability of maternal depression was associated with significantly lower instability in biweekly adolescent sadness (a’ path; *B* = −0.354, SE = 0.155, *β* = −0.122, *p* = 0.022) and, in turn, biweekly instability in adolescent sadness was inversely associated with significantly reduced odds of anxiety (b’ path; OR = 0.556 [0.339, 0.947], *p* = 0.030). However, the indirect effect of variability in maternal depression on young adult anxiety via instability in biweekly adolescent sadness was not statistically significant (*p* = 0.055). Furthermore, there was no evidence of an indirect effect of maternal depression total exposure and instability on young adult depression and anxiety via dynamics of adolescent sadness or happiness.

### Secondary analyses

In the first secondary analysis, the overall pattern of results remained similar when controlling for maternal depression when offspring were between age 9 and 15 and youth internalizing symptoms at age 15. Moreover, results remained consistent when conducted in the primary sample (with 4/7ths of diaries completed) versus full sample. Results indicated that maternal depression total exposure was significantly associated with greater odds of offspring anxiety in young adulthood in Study 1 (OR = 1.27, 95% CI [1.004, 1.60], *p* = 0.046) but not Study 2 (*p* = 0.766). Maternal depression total exposure was not associated with offspring depression in young adulthood in either sample (*p*'s > 0.355), and instability of maternal depression was not significantly associated with offspring depression or anxiety in young adulthood in Study 1 or Study 2 (*p*'s > 0.507). Supporting Information S1: Table S5 presents full results for Aim 1 in the full sample, Study 1, and Study 2.

## DISCUSSION

The present study evaluated the associations of total exposure to and instability of maternal depression across childhood on offspring anxiety and depression in young adulthood and tested adolescent emotion dynamics as novel transdiagnostic pathways. Consistent with the cumulative stress model (Goodman et al., 2020; Shonkoff et al., 2012), greater total exposure to maternal depression was associated with greater odds of offspring depression during young adulthood. Biweekly (but not daily) dynamics of adolescent emotions also predicted young adult anxiety and depression, above and beyond mean levels of emotions. Although instability of maternal depression was not directly associated with young adult depression or anxiety, there was preliminary evidence that greater instability of maternal depression prospectively predicted biweekly dynamics of adolescent sadness, which in turn was associated with young adult anxiety. Together, these results highlight the importance of considering how emotion dynamics across generations and timescales may operate as understudied transdiagnostic risk factors (maternal depression variation from infancy through middle childhood) and mechanisms (biweekly instability in emotional functioning) of young adult psychopathology. These findings require replication in future studies that also examine emotion dynamics across timescales as a transdiagnostic mechanism linking environmental factors to offspring psychopathology, particularly in hypothesis‐driven and theoretically motivated work.

This study is among the first to our knowledge to evaluate antecedents of adolescent emotion dynamics, focusing on both maternal depression total exposure and instability as prospective predictors. Attesting to the importance of considering both temporally sensitive measures of the ebb and flow of emotions and variability, instability (MSSD) in adolescent's sadness was associated with prior maternal depression. Notably, however, the pattern of association varied for total exposure versus instability in maternal depression. Regarding *total* exposure to maternal depression, adolescents who had been more exposed to maternal depression exhibited greater instability of biweekly sadness, extending prior studies that demonstrated positive associations between childhood adversity exposure and variability in adolescent positive emotions (Pelotonen et al., 2024). Depressed mothers may model maladaptive emotion regulation strategies (Buckholdt et al., 2013) and elicit dependent stressful life events (Davis & Glynn, 2024), including greater mother‐child conflict (Brennan et al., 2003; Hammen et al., 2004), that elicit greater instability in adolescent emotions week‐over‐week.

In contrast, *instability* of maternal depression was inversely associated with instability (MSSD) of daily adolescent happiness and variability and instability of biweekly adolescent sadness. In the context of varying levels of maternal depression, after accounting for overall exposure to maternal depression, greater adolescent emotional variability may reflect context‐appropriate adjustment to the ebb and flow of maternal mood symptoms. For example, to brace themselves against unpredictability in maternal depressed mood and behavior, adolescents may dampen natural fluctuations in their emotions, though this may come at the expense of their ability to capitalize on relatively larger changes in emotions (e.g., exceptionally “bright” days or periods of less sadness). Taken together, these findings may suggest adolescents develop patterns of emotion dynamics that may be adaptive within their environmental conditions, consistent with theory and emerging empirical evidence indicating that patterns of maternal depression may have distinct influences on offspring development compared to levels or total exposure to maternal depression (Cents et al., 2012; Davis & Glynn, 2024; Rinne et al., 2022). Notably, the opposite pattern of association between total exposure to maternal depression and instability in maternal depression was only observed for biweekly dynamics of adolescent sadness, not daily emotion dynamics or biweekly dynamics of adolescent happiness. This may suggest that maternal depression total exposure and dynamics have distinct influences on expression of adolescent sadness on a biweekly timescale, potentially because it is a cardinal feature of depression and a 2‐week period is the minimum length for a major depressive episode per DSM criteria (American Psychiatric Association, 2022). However, further research is necessary to determine whether the same pattern would generalize to the dynamics of sadness measured across different timescales (e.g., EMA with fine‐grained assessment of affect multiple times a day), or dynamics of other negative emotions.

At the same time, maternal depression‐related and context‐adapted patterns of emotion dynamics may also confer increased risk for internalizing disorders in young adulthood when offspring are no longer living with their parents or reliant on mothers for emotional support, guidance, and modeling. Contrary to our expectations, lower biweekly instability of sadness was prospectively associated with increased odds of young adult anxiety and may serve as a pathway linking maternal depression variation to the odds of developing anxiety in young adulthood. Similarly, and contrary to prior work (Abitante et al., 2024; Fisher et al., 2025; Houben et al., 2015; Reitsema et al., 2022), greater variability, instability, and inertia of biweekly adolescent happiness were each associated with decreased odds of young adult depression; instability of biweekly happiness was also associated with decreased odds of young adult anxiety. Although in past work excessive fluctuations in emotional experience conferred risk for mental health problems among low‐risk community samples, in the broader context of environmental unpredictability (e.g., neighborhood, household, financial) that disproportionately affects contextually‐disadvantaged lower‐income and racial minority families, more dynamic emotion processes may reflect normative or even adaptive emotional processes during adolescence (Houben et al., 2015; Reitsema et al., 2022). Indeed, past work shows that flexibility, particularly in positive emotional experiences, may be a key source of resilience (Vannucci et al., 2019). Overall, the finding that adolescent emotion dynamics related to subsequent young adult mental health aligns with functional theories of emotion regulation that contend that how emotions unfold over time—above and beyond emotion levels—are a core component of psychological functioning (Shao & Ong, 2026).

Given that we found that biweekly, but not daily, emotion dynamics were associated with young adult internalizing disorders, it is possible that emotion dynamics across timescales (day‐to‐day, week‐to‐week) reflect phenomenologically distinct processes. For example, although there were differences in study administration in Study 1 and Study 2, we found that emotion dynamics involving happiness were not correlated across different timescales (day‐to‐day vs. biweekly; see Supporting Information S1: Table S3) and had differential associations with maternal depression and young adult outcomes. Compared to *daily* emotions, greater variability, instability, and inertia in *biweekly* emotions, may reflect greater emotional and behavioral “flexibility” whereas lower levels of these dynamics may reflect “rigidity,” which is a transdiagnostic feature of common forms of psychopathology (Hollenstein et al., 2004). Consistent with theoretical models of adolescent emotion dynamics (i.e., Flex3), these findings provide evidence that flexibility in both positive and negative emotions at the often overlooked “meso” timescale of biweekly variation may be crucial for mitigating youth internalizing risk (Hollenstein, 2015; Hollenstein et al., 2013). Differences in the timescale may also contribute to the discrepancies between the current study and past research, as past work has examined more fine‐grained variation in emotions multiple times per day (Abitante et al., 2024; Fisher et al., 2025). However, it is also possible that the differences observed in the present study were in part due to differences in the emotions assessed in each study and scale in which they were assessed (e.g., frequency in the biweekly study and intensity in the daily study). Future tests to disentangle whether differences in daily and biweekly study results are due to the timescales examined or the emotions assessed are necessary, as differences in measurement decisions across each diary study precluded our ability to do so here.

### Strengths and limitations

Our study benefited from several strengths, including its longitudinal design, repeated assessment of maternal depression, and relatively large and socioeconomically and racially diverse sample of families (Gelaye et al., 2016; Madigan et al., 2017; Matijasevich et al., 2015; Shakeel et al., 2018). Though often overshadowed by cumulative exposure perspectives, the present findings add to a growing body of evidence showing that variation in maternal depression also uniquely contributes to offspring adjustment (Davis & Glynn, 2024). In addition to contributing to a burgeoning body of work on dynamics of maternal mood and mental health, the present study also assessed variability in adolescent emotions across diverse timescales, including understudied temporally sensitive dynamics of emotional instability and inertia (Reitsema et al., 2022). Notably, the observed pattern of results was robust in secondary analyses, including with conservative control of maternal depression during offspring's adolescence and adolescent concurrent internalizing problems.

At the same time, our results must be viewed in the context of study limitations. Although the study benefited from the use of clinical interviews to assess maternal depression and youth depression and anxiety, even subclinical maternal depressive symptoms (which were not assessed in FFCWS) affect youth development and assessing unpredictability in the severity of maternal depression may have yielded greater power to detect direct effects on youth outcomes (Chiang et al., 2023; Glynn et al., 2018; Goodman, 2020; Winstone‐Weide et al., 2023). Furthermore, the use of diagnostic (binary) measures of maternal depression precluded examination of the full range of measures of dynamics (e.g., variability and inertia). Similarly, the diary substudies were limited by the single‐item assessment of adolescent happiness, in contrast to prior work that assessed multiple positive emotions (e.g., interest, happiness) (Abitante et al., 2024; Fisher et al., 2025). Given the nascent stage of the literature in this research area, the present study did not apply multiple testing corrections to avoid Type 2 errors and maintain statistical power. However, numerous models were conducted across the two studies, which increases the risk of Type I error and should be kept in mind when interpreting the present findings.

Future work with repeated assessment of emotion dynamics in young adulthood is also necessary, as in the approximately 7 years between assessment of adolescent emotion dynamics and young adult mental health, emotion dynamics may have changed, or other factors may be more proximal to young adult mental health. Relatedly, future studies could expand from these findings by testing similar research questions in a causal mediation framework, as the present results preclude any conclusions about causality. Though the present studies benefited from a nationally representative sample, diverse sample, our results may not generalize to other caregivers (e.g., fathers, grandparents) or families (e.g., rural communities, non‐Western countries) and there may be differences in the observed associations depending on sociodemographic factors. Finally, despite review evidence that contributions of maternal depression to child development are independent of genetic confounding (Natsuaki et al., 2014), the present study did not control for genetic confounding, and shared genetic influences could impact the results observed here.

### Conclusion

The goal of the present study was to test the prospective associations of maternal depression total exposure and instability with offspring internalizing disorders during young adulthood and investigate offspring emotion dynamics as potential mechanistic pathways. Results highlighted distinct associations of maternal depression total exposure and instability on both offspring emotion dynamics, depression, and anxiety. Findings also showed, contrary to hypotheses, that greater variability, instability, and inertia in adolescent emotion dynamics reduced the likelihood of internalizing disorders in young adulthood. Collectively, these results underscore the importance of considering the influence of dynamic variation in mood and emotions, across generations and timescales, on young adult psychopathology risk.

## AUTHOR CONTRIBUTIONS

**Gabrielle R. Rinne:** Conceptualization; writing—original draft; writing—review and editing; methodology; formal analysis. **Blakely L. Berryhill:** Writing—original draft; writing—review and editing; formal analysis. **Caroline M. Chandler:** Writing—original draft; writing—review and editing. **Jennifer A. Somers:** Writing—original draft; writing—review and editing; formal analysis; conceptualization; data curation; funding acquisition.

## CONFLICT OF INTEREST STATEMENT

The authors declare no conflicts of interest.

## ETHICAL CONSIDERATIONS

All participants provided informed consent. Study protocol for FFCWS and each substudy was approved by IRB at each study site the study was conducted (Princeton University, Columbia University, and Brigham Women's Hospital IRB approved the FFCWS study protocol; Princeton University and Stony Brook University IRB approved the sleep substudy; Princeton University IRB approved the mDiary substudy). The IRB approval/reference numbers and approval dates are not available publicly or to the present study authors; each can be obtained directly from the FFCWS study personnel.

## Supporting information

Supporting Information S1

## Data Availability

The data that support the findings of this study are available on request from FFCWS Study Team at (https://ffcws.princeton.edu/).

## Endnote

^1^
Covariate effects in primary analyses are described in the Supporting Information file, Supporting Information S1: Appendix S4.
