# Supplementary material for: Maternal depression across childhood and offspring young adult depression and anxiety: Testing adolescent emotion dynamics as transdiagnostic mechanisms
Source: JCPP Adv. 2026 May 4:e70130. Online ahead of print. doi: 10.1002/jcv2.70130 (PMC13339266; doi:10.1002/jcv2.70130)
Supplement: Supplementary file 1 — Supporting Information S1 [file JCV2-9999-e70130-s001.docx]

**Maternal Depression Across Childhood and Offspring Young Adult Depression and Anxiety: Testing Adolescent Emotion Dynamics as Transdiagnostic Mechanisms**

**Supporting Information**

| **Table S1.** Maternal Depression Total Exposure Categories | |
| --- | --- |
|  | **% (*n*)** |
| 1. Always not depressed | 35.7% (1750) |
| 1. Not depressed whenever known | 25.2% (1236) |
| 1. Not depressed 3x, depressed 1x | 20.1% (982) |
| 1. Not depressed ≥1x, depressed ≥2x | 11.8% (576) |
| 1. Always depressed when known (but not always known) | 1.5% (71) |
| 1. Always depressed | 1.9% (91) |
| *Always missing | 3.9% (192) |
| Total | 4898 (100%) |
| *Note.* *Participants who were missing maternal depression across all timepoints were not included in primary analyses. This approach was informed by past work in the FFCWS study (see Geller & Curtis, 2018). | |

**Appendix S1. Deviations from Preregistration**

Deviations from the preregistration were due to data availability and characteristics of the data unknown until data access/following preregistration (see Table S2 below for a summary). Though we originally intended to assess youth internalizing symptom severity (based on BDI and BAI), this information was only available for a small subsample of the FFCWS participants (as part of the SAND neuroimaging substudy); thus, we used CIDI-SF data, which was available for the entire sample at year 22. We also originally intended to conduct a Latent Class Analysis to identify distinct classes of maternal depression; however, model fit was poor and there were issues with convergence, as is not uncommon for Latent Class Analysis with binary indicators (Nylund-Gibson & Choi, 2018). We also considered maternal depression between ages 9-15 years as a covariate; this was not included in our initial list of potential covariates in the preregistration, as we did not know this data was available at the time of our preregistration. In addition, although we planned to use the parent-reported pubertal development scale data, low internal consistency (alpha = 0.16 for girls; alpha = 0.01 for boys) precluded this.

| **Table S2.** Summary of Deviations from Pre-Registration | | | | |
| --- | --- | --- | --- | --- |
| **Deviations** | | | | |
| **No.** | **Details** | **Original Wording** | **Deviation Description** | **Reader Impact** |
| 1 | Type: Variables  Reason: Plan not possible  Timing: After data access | “Depression and anxiety symptoms were assessed with the Beck Depression Inventory (BDI) and Beck Anxiety Inventory (BAI), respectively, at child age 22.” | BDI and BAI measures were not available in the full sample; therefore, we used CIDI-SF data as the primary young adult outcome variable. | Primary young adult outcomes of depression and anxiety are based on CIDI-SF, not BDI and BAI scores. |
| 2 | Type: Analysis  Reason: Plan not possible  Timing: After data access | “We will conduct a latent class analysis to test Hypothesis 1”  “If our latent class model of maternal depression trajectories will not converge, we will adopt the approach to identifying trajectories of housing insecurity used in Geller & Curtis (2018).” | The latent class analyses of maternal depression did not converge. Thus, as outlined in the preregistration, we used a descriptive approach to classify instability of maternal depression. | Results are presented for a descriptive approach to characterizing maternal depression instability. |
| 3 | Type: Variables  Reason: New knowledge  Timing: After data access | “We will also assess potential confounds and potential correlates of missingness: from baseline (birth) survey: mother and child race and ethnicity, maternal country of origin, child biological sex, household material hardship (poverty ratio), maternal education, maternal marital status, maternal age; pubertal development (year 9), and prior adolescent (year 15) depressive and anxiety (internalizing) symptoms (CBCL; Archenbach, 1992).” | We did not adjust for pubertal status due to low reliability of pubertal status measures in FFCWS. | The primary results presented did not adjust for pubertal status. |
| **Unregistered Steps** | | | | |
| **No.** | **Details** | **Original Wording** | **Deviation Description** | **Reader Impact** |
| 1 | Type: Variables  Reason: New knowledge  Timing: After data access | Not applicable. | Maternal depression from child age 9-15 was not initially included in the list of covariates because we were not aware the variable was available until after data access. | Secondary analyses include maternal depression from child age 9-15 as a covariate. There is no impact on the primary analyse |
| *Note.* This table follows recommendations for reporting deviations from pre-registrations outlined in Willroth et al., 2024 (<https://doi.org/10.1177/25152459231213802>). | | | | |

**Appendix S2. Emotion Dynamics Preliminary Analyses**

We first evaluated whether there were linear trends in each affect time series, pooled across the full sample. In Study 1, there was a statistically significant linear decrease in happiness, Est = -0.059, SE Est = 0.008, *p* < .001; but no significant change in loneliness or anger (*p*’s > 0.70). In Study 2, there was a statistically significant linear decrease in sadness, Est = -0.004, SE Est = 0.001, *t*(9278) = -3.17, *p* = 0.0016, and a statistically significant linear increase in happiness, Est = 0.006, SE Est = 0.001, *t*(9286) = 4.04, *p* < .001. The happiness time series from Study 1 and Study 2 and the sadness time series from the Study 2 were detrended by obtaining the residuals of the affect data from the equation predicting the affect variable from linear trend; affect variability and instability were then calculated on the residualized data.

Emotion dynamics in the mDiary study were positively correlated within and across positive (happy) and negative affect (sad), *r*’s: 0.11-0.96, *p*’s < .001, with the exception that the mean of happiness and sadness were negatively correlated, *r* = -.374, *p* < .001. Emotion dynamics in the sleep study were generally positively correlated (except for iSD of happy with the MSSD and inertia of happiness), though the strength and significance of associations varied within and across constructs. Dynamics involving happiness obtained from the daily diaries in the sleep study were not correlated with dynamics involving happiness obtained from the biweekly diaries in the mDiary study (*p*’s > .05). Daily dynamics involving anger and loneliness were positively correlated with several measures of biweekly dynamics in adolescent sadness. Table S2 presents bivariate associations of emotion dynamics measures across Study 1 and Study 2.

| **Table S3.** Bivariate associations of emotion dynamics and levels measures across studies Study 1 (daily) and Study 2 (biweekly) | | | | | | | | | | | | | |
| --- | --- | --- | --- | --- | --- | --- | --- | --- | --- | --- | --- | --- | --- |
|  |  | **iSD Happy Daily** | **iSD Angry Daily** | **iSD Lonely Daily** | **MSSD Happy**  **Daily** | **MSSD Angry Daily** | **MSSD Lonely Daily** | **Inertia Happy Daily** | **Inertia Angry Daily** | **Inertia Lonely Daily** | **Mean Happy Daily** | **Mean Angry Daily** | **Mean Lonely Daily** |
| **iSD Sad Biweekly** | *r* | -0.140 | 0.171 | 0.204 | 0.042 | 0.071 | 0.153 | 0.133 | 0.107 | 0.061 | 0.083 | 0.175 | 0.121 |
|  | *p*-value | 0.093 | 0.028 | 0.008 | 0.620 | 0.373 | 0.055 | 0.073 | 0.162 | 0.423 | 0.321 | 0.021 | 0.109 |
| **MSSD Sad Biweekly** | *r* | -0.063 | 0.122 | 0.198 | 0.012 | 0.032 | 0.179 | 0.074 | 0.107 | 0.085 | 0.105 | 0.168 | 0.104 |
|  | *p*-value | 0.452 | 0.119 | 0.010 | 0.883 | 0.686 | 0.024 | 0.322 | 0.160 | 0.262 | 0.208 | 0.027 | 0.167 |
| **Inertia Sad Biweekly** | *r* | -0.009 | 0.083 | 0.163 | 0.014 | 0.040 | 0.148 | 0.059 | 0.073 | 0.110 | 0.129 | 0.128 | 0.045 |
|  | *p*-value | 0.911 | 0.246 | 0.022 | 0.858 | 0.582 | 0.042 | 0.389 | 0.300 | 0.114 | 0.093 | 0.067 | 0.513 |
| **Mean Sad Biweekly** | *r* | 0.002 | 0.061 | 0.097 | 0.085 | 0.038 | 0.235 | 0.111 | 0.040 | 0.001 | 0.078 | -0.015 | 0.096 |
|  | *p*-value | 0.982 | 0.436 | 0.215 | 0.310 | 0.633 | 0.003 | 0.133 | 0.602 | 0.992 | 0.352 | 0.846 | 0.203 |
| **iSD Happy Biweekly** | *r* | -0.145 | 0.122 | 0.060 | -0.049 | 0.052 | 0.074 | 0.067 | 0.050 | 0.001 | 0.100 | 0.082 | 0.048 |
|  | *p*-value | 0.083 | 0.116 | 0.443 | 0.557 | 0.511 | 0.348 | 0.365 | 0.516 | 0.989 | 0.231 | 0.281 | 0.523 |
| **MSSD Happy Biweekly** | *r* | 0.054 | 0.013 | 0.131 | -0.087 | -0.078 | 0.195 | 0.016 | 0.099 | 0.016 | 0.090 | 0.154 | 0.077 |
|  | *p*-value | 0.523 | 0.868 | 0.090 | 0.303 | 0.328 | 0.013 | 0.834 | 0.196 | 0.837 | 0.283 | 0.043 | 0.306 |
| **Inertia Happy Biweekly** | *r* | 0.075 | 0.004 | 0.064 | -0.062 | -0.085 | 0.118 | 0.030 | 0.082 | 0.016 | 0.036 | 0.084 | 0.020 |
|  | *p*-value | 0.331 | 0.960 | 0.371 | 0.426 | 0.242 | 0.106 | 0.660 | 0.241 | 0.822 | 0.642 | 0.230 | 0.771 |
| **Mean Happy Biweekly** | *r* | 0.065 | -0.143 | -0.189 | -0.043 | -0.139 | -0.267 | -0.054 | -0.101 | -0.016 | -0.101 | -0.062 | -0.039 |
|  | *p*-value | 0.437 | 0.065 | 0.014 | 0.612 | 0.078 | <.001 | 0.465 | 0.185 | 0.831 | 0.226 | 0.418 | 0.608 |
| *Note.* Blue highlight indicates a significant positive association. Green highlight indicates a significant negative association. | | | | | | | | | | | | | |

**Appendix S3. Covariate Selection**

The following variables from the FFCWS baseline survey were considered as potential covariates: maternal race, geographical region of mother’s birth place, child biological sex, poverty ratio, maternal education, mother’s relationship status with the child’s biological father, and maternal age. In addition, the following variables from the year-15 follow-up wave survey were considered as potential covariates: whether the primary caregiver was the child’s biological mother at year 15 and the child’s pubertal development. The number of surveys completed was also considered as a covariate of emotion dynamics. We also considered time (in months) since March 2020 as a potential covariate of the Year 22 mental health outcomes.

Covariates were selected based on their associations with levels of and/or missingness on primary outcomes (either emotion dynamics at year 15 and/or anxiety or depression diagnoses at year 22) (*p*’s < 0.05). Maternal race was associated with missingness on sleep (daily) diary emotion dynamics, inertia of anger in the sleep (daily) diary substudy, and youth anxiety and depression diagnoses. Paternal race was associated with sleep (daily) diary emotion dynamics and youth anxiety and depression diagnoses. Child biological sex was associated with intraindividual variability of sadness in the mDiary (biweekly) substudy; intraindividual variability of anger and loneliness, instability and inertia of anger, and mean of loneliness and anger in the sleep (daily) diary substudy, and youth and anxiety depression diagnoses. Baseline household poverty ratio was associated with the intraindividual standard deviation of sadness and the intraindividual standard deviation of happiness in the biweekly and daily diary substudies, respectively, as well as with youth depression diagnosis. Maternal education was related to missingness on emotion dynamics in both substudies, and to the instability of sadness in the biweekly substudy and the intraindividual standard deviation of happiness in the daily diary substudy, and to youth depression diagnoses. Maternal marital status at baseline was also associated with missingness on emotion dynamics in both substudies.

Maternal depression diagnosis between child ages 9-15 years was associated with instability of happiness in the daily diary substudy and youth depression and anxiety diagnoses. Youth internalizing problems at 15 years were associated with the intraindividual standard deviation, instability, and mean of anger and loneliness in the daily diary substudy, as well as youth depression and anxiety diagnoses.

**Appendix S4. Covariate Effects in Primary Analyses**

For ease of reading and due to space constraints, covariate effects are not presented in the main text. In Study 1, girls were significantly more likely to have depression or anxiety in young adulthood compared to boys and offspring of Black, Non-Hispanic mothers were more likely to have depression in young adulthood. In Study 2, mean levels of loneliness and anger were significantly associated with greater odds of young adult depression and anxiety. Mean levels of happiness were also associated with greater odds of young adult anxiety. Girls were also significantly more likely to have depression or anxiety in young adulthood compared to boys.

| **Table S4.** Maternal depression, emotion dynamics, and offspring depression and anxiety in young adulthood. | | | | | | |
| --- | --- | --- | --- | --- | --- | --- |
| **Full Sample** |  |  |  |  |  |  |
|  | **No Anxiety** **(87.3%)** | **Anxiety** **(12.7%)** |  | **No Depression** **(61.4%)** | **Depression (38.6%)** |  |
| **Maternal Depression** | ***M (SD)*** | ***M (SD)*** | ***t*-statistic** | ***M (SD)*** | ***M (SD)*** | ***t*-statistic** |
| Total Exposure | 2.11 (1.37) | 2.33 (1.27) | 2.77** | 2.07 (1.27) | 2.26 (1.31) | -4.03*** |
| Instability | 0.19 (0.29) | 0.24 (0.36) | -2.34 | 0.18 (0.28) | 0.23 (0.30) | -3.75*** |
| **Study 1** |  |  |  |  |  |  |
|  | **No Anxiety (88.1%)** | **Anxiety (11.9%)** |  | **No Depression** **(61.4%)** | **Depression** **(38.6%)** |  |
| **Daily Emotion Dynamics** | ***M (SD)*** | ***M (SD)*** | ***t*-statistic** | ***M (SD)*** | ***M (SD)*** | ***t*-statistic** |
| Happy iSD | 1.10 (0.06) | 1.10 (0.06) | 0.25 | 1.10 (0.06) | 1.10 (0.06) | 0.08 |
| Happy MSSD | 2.80 (1.06) | 2.87 (1.14) | -0.49 | 2.82 (1.09) | 2.78 (0.96) | 0.58 |
| Happy Inertia | 0.21 (0.32) | 0.29 (0.34) | -0.82^ | 0.22 (0.33) | 0.23 (0.31) | -0.52 |
| Angry iSD | 0.57 (0.56) | 0.77 (0.48) | -2.84** | 0.53 (0.53) | 0.72 (0.57) | -4.29*** |
| Angry MSSD | 1.20 (2.00) | 1.47 (1.90) | -1.06 | 1.10 (1.92) | 1.54 (2.25) | -2.68** |
| Angry Inertia | 0.15 (0.31) | 0.26 (0.32) | -2.84** | 0.15 (0.32) | 0.19 (0.29) | -1.85^ |
| Lonely iSD | 0.44 (0.52) | 0.75 (0.60) | -4.46*** | 0.42 (0.52) | 0.57 (0.56) | -3.62*** |
| Lonely MSSD | 0.83 (1.67) | 1.66 (2.53) | -3.46*** | 0.82 (1.91) | 1.06 (1.67) | -1.67^ |
| Lonely Inertia | 0.14 (0.29) | 0.26 (0.35) | -3.11** | 0.13 (0.29) | 0.18 (0.31) | -2.34* |
| **Study 2** |  |  |  |  |  |  |
|  | **No Anxiety (82.5%)** | **Anxiety (17.5%)** |  | **No Depression** **(54.2%)** | **Depression** **(45.8%)** |  |
| **Biweekly Emotion Dynamics** | ***M (SD)*** | ***M (SD)*** | ***t*-statistic** | ***M (SD)*** | ***M (SD)*** | ***t*-statistic** |
| Sad iSD | 1.04 (0.04) | 1.04 (0.03) | 0.23 | 1.05 (0.04) | 1.03 (0.04) | 1.59 |
| Sad MSSD | 2.04 (0.68) | 1.85 (0.82) | 1.93^ | 2.07 (0.70) | 2.00 (0.75) | 0.99 |
| Sad Inertia | 0.01 (0.25) | -0.05 (0.31) | 1.57 | 0.02 (0.25) | -0.003 (0.28) | 1.00 |
| Happy iSD | 1.04 (0.05) | 1.04 (0.05) | 0.44 | 1.04 (0.05) | 1.04 (0.04) | 1.51 |
| Happy MSSD | 2.08 (0.77) | 1.92 (0.68) | 1.51 | 2.14 (0.77) | 1.94 (0.73) | 2.58* |
| Happy Inertia | 0.01 (0.26) | -0.02 (0.25) | 1.13 | 3.90 (2.62) | -3.49 (2.62) | 3.13** |
| *Note.* *** *p* < .001; ** *p* < .01; * *p* < .05; ^ p < .10. iSD = Variability; MSSD = Instability. | | | | | | |

| **Table S5.** Aim 1 Results in Full Sample, Study 1, and Study 2 | | | | |
| --- | --- | --- | --- | --- |
|  | **Depression** | | **Anxiety** | |
|  | **OR [95% CI]** | ***p*-value** | **OR [95% CI]** | ***p*-value** |
| **Full Sample** |  |  |  |  |
| Maternal Depression: Total Exposure | 1.10 [1.01, 1.20] | .025* | 1.13 [1.00, 1.29] | .062^ |
| Maternal Depression: Instability | 1.40 [0.93, 2.12] | .107 | 1.21 [0.64, 2.26] | .556 |
| **Study 1** |  |  |  |  |
| Maternal Depression: Total Exposure | 1.08 [0.92, 1.28] | .355 | 1.27 [1.004, 1.60] | .046* |
| Maternal Depression: Instability | 1.17 [0.54, 2.52] | .696 | 0.84 [0.28, 2.52] | .761 |
| **Study 2** |  |  |  |  |
| Maternal Depression: Total Exposure | 1.03 [0.82, 1.30] | .782 | 0.96 [0.71, 1.29] | .766 |
| Maternal Depression: Instability | 1.41 [0.45, 4.35] | .555 | 1.61 [0.40, 6.51] | .507 |
| *Note.* OR = odds ratio. CI = confidence interval.  * *p* < .05; ^ *p* < .10. | | | | |
